# Using Video-Assisted Laryngoscope (GlideScope®) to Insert a Nasogastric Tube and Prevent Pneumothorax From Incorrectly Inserted Nasogastric Tubes

**DOI:** 10.7759/cureus.9720

**Published:** 2020-08-13

**Authors:** Oday Z AlHafidh, Danilo Enriquez, Joseph Quist, Frances Schmidt

**Affiliations:** 1 Pulmonary, Interfaith Medical Center, Brooklyn, USA; 2 Pulmonary Medicine, Interfaith Medical Center, Brooklyn, USA

**Keywords:** nasogastric tube (ngt), video-assisted laryngeoscope (val), chest x-ray (cx-ray), pneumothorax (ptx)

## Abstract

When indicated, nasogastric tubes (NGT) are often inserted blindly, and the positioning is later confirmed using a chest X-ray (CX-ray). This case describes the blind insertion of an NGT in an 85-year-old nonverbal woman with advanced dementia who developed a pneumothorax (PTX) following NGT insertion. The patient had sepsis due to pneumonia and an infected decubitus ulcer. Because the patient had difficulty swallowing, NGT insertion was blindly performed by a house staff resident, and the tube entered the left lung. A portable bedside CX-ray was performed post-insertion and confirmed that the NGT was in the left lung, with left-sided PTX. An immediate CT of the chest revealed a partial collapse of the left lung. The patient was placed on a nonrebreather mask with 80% oxygen, and immediate insertion of a chest tube (12 Fr catheter) resulted in a subcomplete resolution of the PTX on the left side, with remaining apical PTX. Because an NGT was still required to feed the patient, we used a video-assisted laryngoscope (VAL) to assist with the insertion of the NGT the second time and prevent insertion in the incorrect location. We encourage physicians to consider the insertion of NGT under direct observation using VAL.

## Introduction

Nasogastric tubes (NGT) are often inserted in intensive care settings, the emergency department, and hospital wards, and usually, the insertion is blindly performed, but the position of the NGT is later confirmed with a chest X-ray (CX-ray) [1]. NGT use is a palliative care option to decompress the bowel in the event of bowel obstruction, and for intubated patients, NGT use helps prevent aspiration when delivering enteral nutrition [2].

We present a case of pneumothorax (PTX) following NGT insertion in an 85-year-old, nonverbal woman with advanced dementia who produced an uncommon complication from a very common procedure. The NGT was properly inserted under direct vision with the assistance of video-assisted laryngoscopy (VAL).

## Case presentation

An 85-year-old woman from a nursing home who had a past medical history of end-stage renal disease on hemodialysis, type 2 diabetes, hypertension, coronary artery disease, Alzheimer’s dementia, and deep vein thrombosis transferred to our facility from her nursing home due to desaturation. A CT angiography (CTA) was performed, and pulmonary embolism was not found. The patient was then diagnosed with sepsis due to pneumonia and an infected decubitus ulcer.

**Figure 1:**
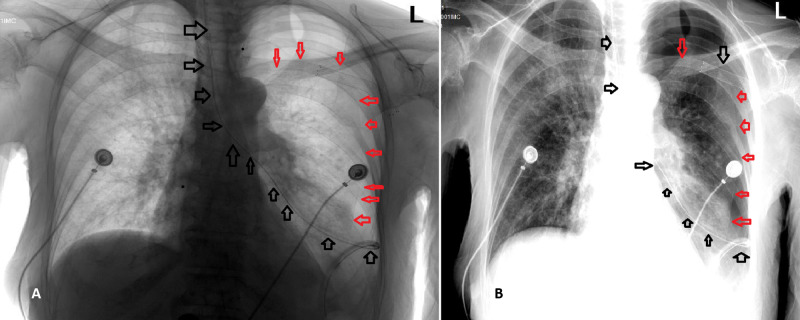
CX-ray immediately after the incorrect placement of NGT Image A shows the inverse mode, and image B shows the regular mode of CX-ray immediately after the incorrect placement of NGT (black arrows), and the development of left-sided PTX (red arrows). CX-ray, chest X-ray; NGT, nasogastric tube; PTX, pneumothorax.

The patient was nonverbal as a baseline, and because she had difficulty in swallowing, it was necessary to initiate feeding with an NGT. A house staff resident blindly performed the NGT insertion, and the tube entered the patient’s left lung. A portable bedside CX-ray confirmed the incorrect NGT placement in the left main bronchus that resulted in left-sided PTX (Figure 1).

**Figure 2:**
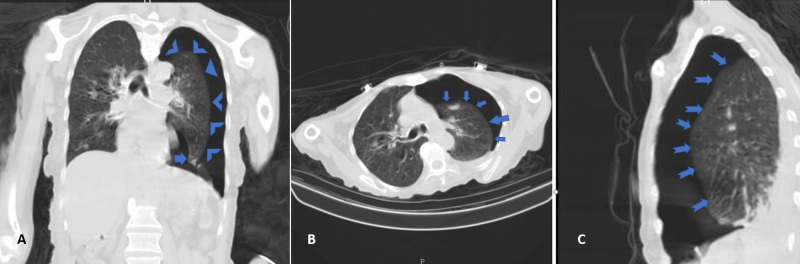
CT chest after removal of the incorrectly placed NGT (A) Coronal plane, (B) axial plane, and (C) sagittal plane CT chest after removal of the incorrectly placed NGT, showing the extent of left-sided PTX. NGT, nasogastric tube; PTX, pneumothorax.

CT of the chest revealed a partial collapse of the left lung (Figure 2), confirming left-sided PTX secondary to NGT placement.

**Figure 3:**
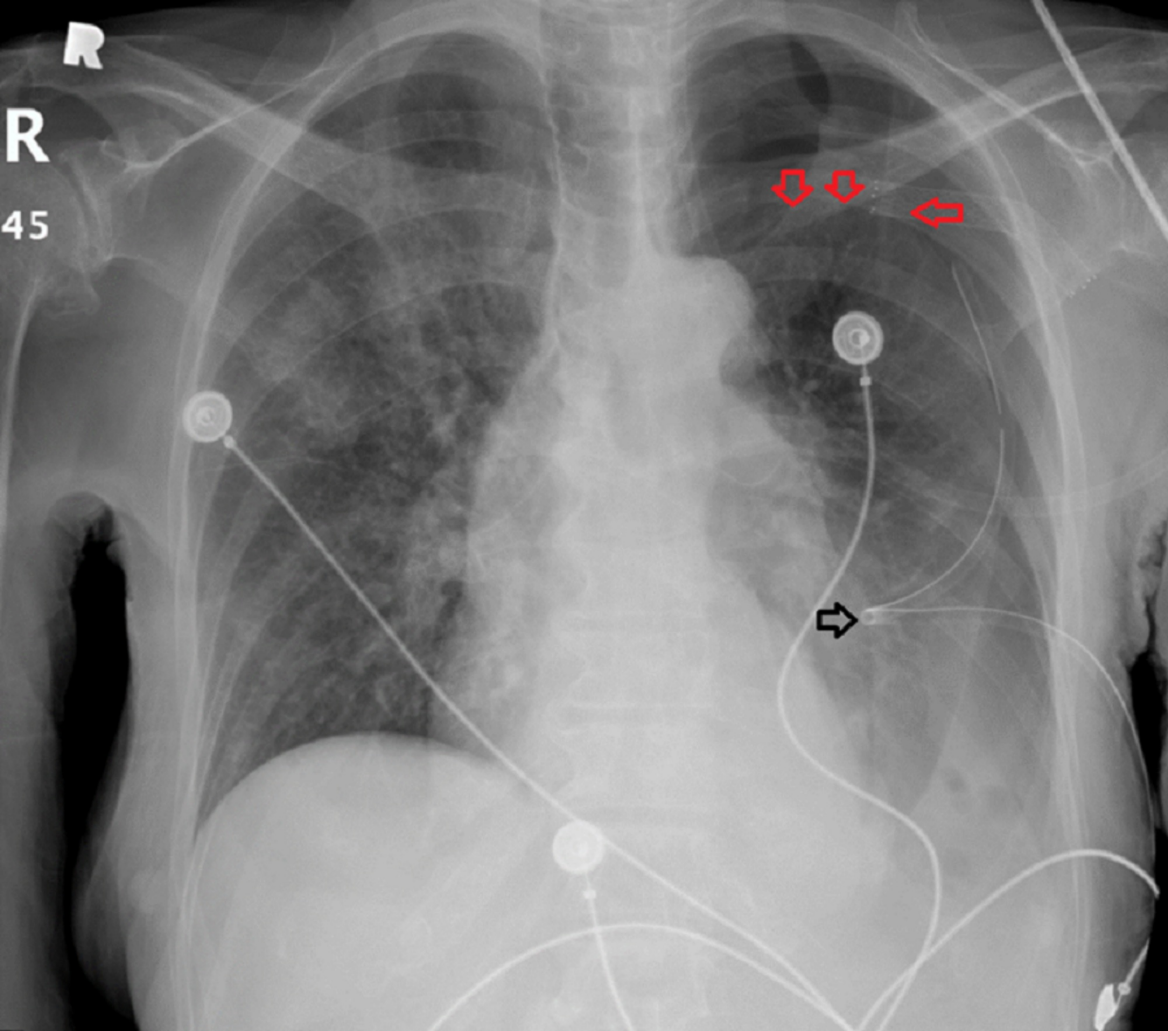
Chest X-ray taken after the first chest tube placement Placement of first chest tube (black arrow), and incomplete resolution of pneumothorax (red arrows).

The patient was placed on oxygen with a nonrebreather mask, on 15 L/minute, and with 80% fraction of inspired oxygen. Immediate insertion of a chest tube (12 Fr catheter) resulted in a subcomplete resolution of the PTX on the left side, with remaining apical PTX. The CX-ray taken after the first chest tube placement revealed remaining apical PTX and suboptimal expansion (Figure 3).

**Figure 4:**
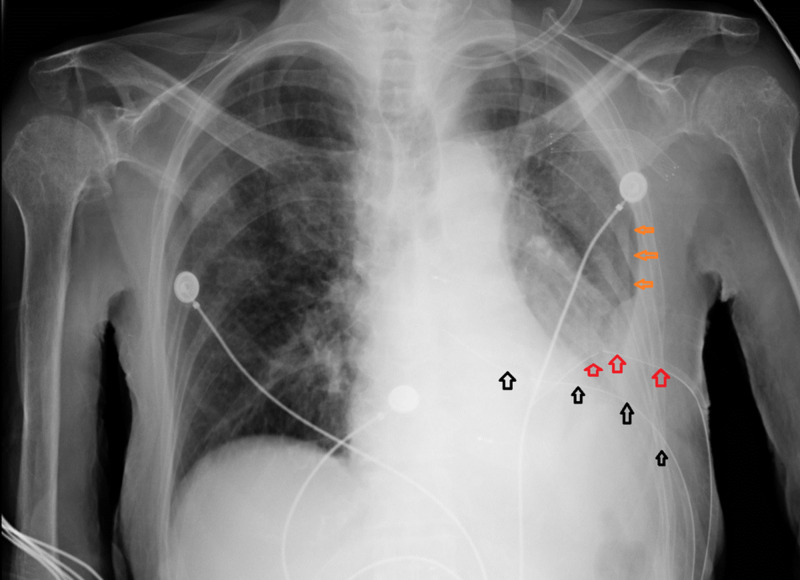
Placement of the second chest tube Placement of the second chest tube (red arrows), with the first chest tube (black arrows), as there was incomplete resolution of pneumothorax (orange arrows).

The next day, it was necessary to place another chest tube due to the incomplete resolution of the left-sided PTX. The chest tubes were set to intermittent wall suction. The CX-ray after the second chest tube placement shows almost complete resolution of left-sided PTX (Figure 4).

After complete resolution of the PTX, the two chest tubes were removed in a stepwise manner over three days, and an additional CX-ray was obtained.

**Figure 5:**
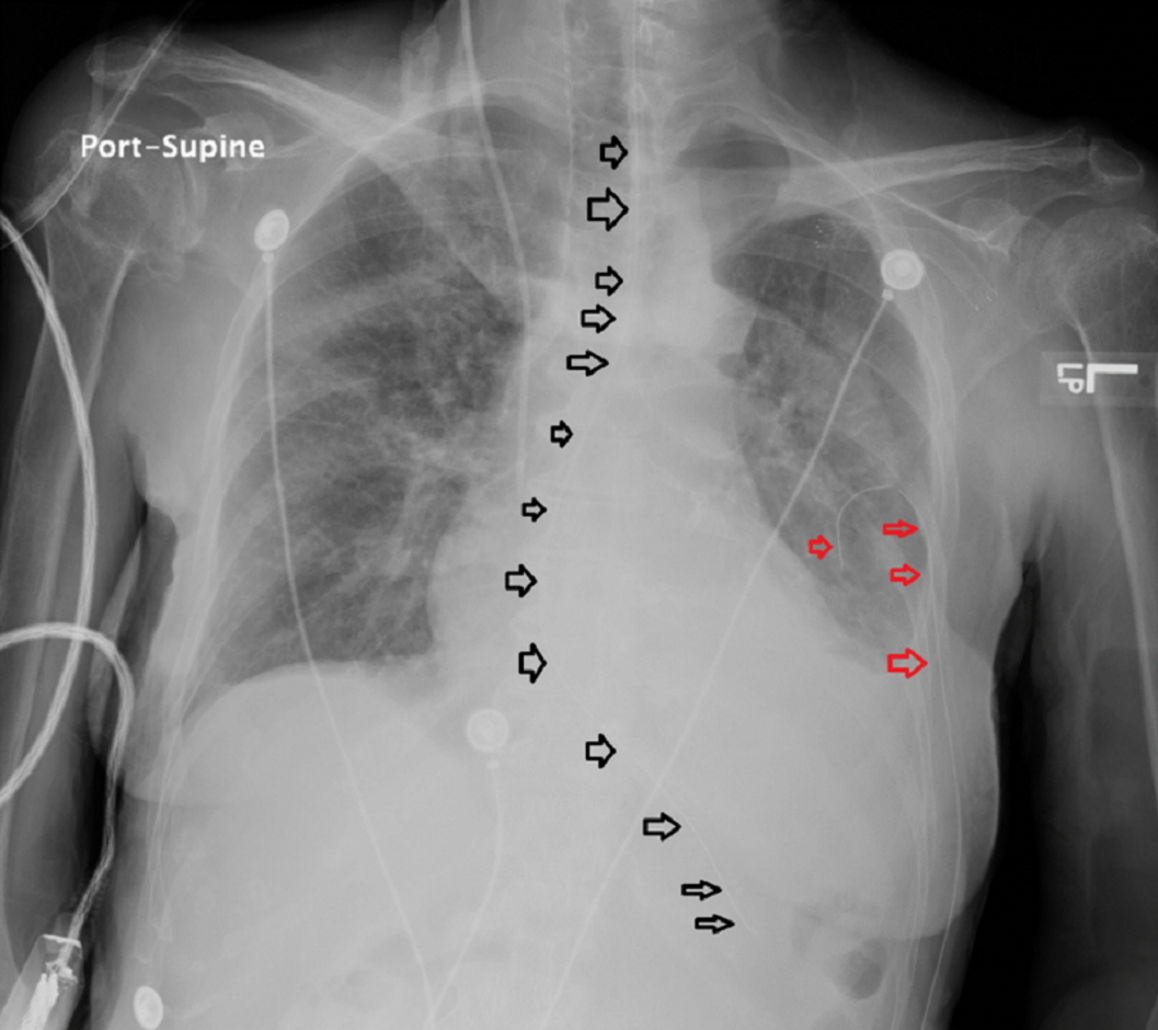
CX-ray after removal of first chest tube, which illustrates the successful placement of the NGT using VAL CX-ray after removal of first chest tube showing second chest tube (red arrows), and correct placement of NGT (black arrows), under direct vision through VAL (GlideScope). CX-ray, chest X-ray; NGT, nasogastric tube; VAL, video-assisted laryngoscopy.

The patient still required an NGT for feeding, and therefore, VAL (GlideScope®, Verathon Inc., Bothell, WA) was used to prevent incorrect insertion of the NGT. After the NGT was placed, a CX-ray was performed (Figure 5).

After proper insertion into the stomach, enteral feeding was successful. The patient later finished the treatment course and eventually was discharged back to the nursing home.

## Discussion

NGT is used mainly in clinical practice to provide enteral nutrition and decompress the gastrointestinal tract in cases of intestinal obstruction. NGT insertion is typically performed by nursing staff or house staff blindly, and most NGTs are successfully placed. However, misplacement can happen, and when it does, it usually occurs in older, debilitated patients or in young patients with fractures at the base of the skull. Both patient types lack a proper gag reflex, and the NGT usually goes into the respiratory tract, leading to serious consequences like PTX, pneumonia, atelectasis, emphysema, bronchopleural fistula, and even death [3].

Other complications from NGT placement have been described, including epistaxis, retropharyngeal dissection, turbinectomy, lung perforation, PTX, pneumomediastinum, esophageal and gastric perforation, rupture of varices, erosion of the nose and/or soft palate, and intracranial intubation [4].

In 2% of the cases, the feeding tube was inserted into the trachea-pulmonary system [5]. Such cases usually occur when the patient lacks a gag reflex, which can occur in debilitated patients or patients with a fracture at the base of the skull. Inadvertent intracranial placement of NGTs may occur during the intubation of patients with severe maxillofacial trauma or basal skull fractures [6]. Repeated malposition in the same patient is common, with 32% of patients who had one intrabronchial misplacement ultimately undergoing multiple NGT misplacements [7].

Between 2005 and 2011, the National Patient Safety Agency was notified of 21 deaths and 79 cases of harm due to misplaced NGTs. It was determined that the single greatest cause of harm was due to misinterpretation of X-rays during the insertion process, accounting for approximately half of all incidents and deaths [8].

The first and most common method for confirming NGT placement is by stethoscope. However, 20% false gastric confirmation by auscultation has been reported [9]. Several other techniques and measures have been used, such as the SORT maneuver, which is the mnemonic for the four main steps constituting the technique: sniffing position, NGT orientation, contralateral rotation, and twisting movement [10].

Capnography is another method that can be used for NGT placement confirmation. After a 30-cm length of feeding tube has been inserted and before the first CX-ray is taken, an end-tidal carbon dioxide detector is attached to the proximal end of the feeding tube. It is left in place for one minute and then observed for a change in color. Originally purple, it will turn tan or even yellow on contact with carbon dioxide. If the end-tidal carbon dioxide detector remains purple, this indicates the gastrointestinal placement of the tube; if it turns tan or yellow, this indicates airway placement of the tube [11].

After following a two-step protocol proposed by Roubenoff and Ravich comprising radiographic confirmation of the tube after blind advancement to 30 cm and another radiograph after advancing to the desired length, there was decreased incidence of PTX caused by NGT insertion [12]. However, this procedure failed to gain widespread acceptance due to its time-consuming nature and lack of cost-effectiveness.

We used a GlideScope VAL, and compared with direct laryngoscope placement, the GlideScope was comparable to the use of the MacIntosh (Grafco, GF Health Products, Inc., Atlanta, GA) laryngoscope in terms of successful rate of insertion and complications [13]. However, a study performed by Moharari et al. showed that less time was required for NGT insertion using the GlideScope [14].

## Conclusions

The use of a VAL (GlideScope) can be less time consuming, with less radiation exposure, and will guarantee safe and direct placement of an NGT in nonintubated patients with a poor gag reflex. For our patient, we used a Glidescope VAL to insert the second NGT, and the process was rapid and successfully completed. We circumvented the use of other uncertain and time-consuming methods for ascertaining the correct path and were able to avoid further complications. Therefore, using a VAL (GlideScope) to directly visualize the path of NGT insertion will facilitate the procedure for the provider and prevent unnecessary and sometimes deadly patient outcomes.
